# Ethics issues experienced in HBM within Portuguese health surveillance and research projects

**DOI:** 10.1186/1476-069X-7-S1-S5

**Published:** 2008-06-05

**Authors:** M Fátima Reis, Susana Segurado, Ana Brantes, Helena Teresinha Simões, J Maurício Melim, V Geraldes, J Pereira Miguel

**Affiliations:** 1 Institute of Preventive Medicine, Faculty of Medicine, University of Lisbon, Av. Prof. Egas Moniz, 1649-028 Lisbon, Portugal; 2 Dr Alfredo da Costa Maternity, Rua Viriato, 1069-089 Lisbon, Portugal; 3 Regional Department of Public Health, Rua das Pretas, 9004-151 Funchal, Portugal

## Abstract

**Background:**

In keeping with the fundamental practice of transparency in the discussion and resolution of ethics conflicts raised by research, a summary of ethics issues raised during Portuguese biomonitoring in health surveillance and research is presented and, where applicable, their resolution is described.

**Methods:**

Projects underway aim to promote the surveillance of public health related to the presence of solid waste incinerators or to study associations between human exposure to environmental factors and adverse health effects. The methodological approach involves biomonitoring of heavy metals, dioxins and/or other persistent organic pollutants in tissues including blood, human milk and both scalp and pubic hair in groups such as the general population, children, pregnant women or women attempting pregnancy. As such, the projects entail the recruitment of individuals representing different demographic and health conditions, the collection of body tissues and personal data, and the processing of the data and results.

**Results:**

The issue of autonomy is raised during the recruitment of participants and during the collection of samples and data. This right is protected by the requirement for prior written, informed consent from the participant or, in the case of children, from their guardian. Recruitment has been successful, among eligible participants, in spite of incentives rarely being offered. The exception has been in obtaining guardians' consent for children's participation, particularly for blood sampling. In an attempt to mitigate the harm-benefit ratio, current research efforts include alternative less invasive biomarkers.

Surveys are currently being conducted under contract as independent biomonitoring actions and as such, must be explicitly disclosed as a potential conflict of interests. Communication of results to participants is in general only practised when a health issue is present and corrective action possible. Concerning human milk a careful approach is taken, considering breast-feeding's proven benefits.

**Conclusion:**

No national legislation currently accounts for the surveillance component of biomonitoring as distinct from research. Ethics issues arising within the domain of research are resolved according to available regulations. For issues encountered during surveillance, the same principles are used as guidance, completed by the authors' best judgement and relevant ethics committees' findings.

## Background

Transparency is of fundamental importance in the discussion and resolution of ethics problems raised within scientific practice, besides being an ethical requirement itself. It is important that ethical challenges, solutions and evaluation of those solutions are documented [1], not only as an ethical responsibility [2], but also in order to be able to refute accusations of improper conduct and to inform other researchers facing the same issues in similar or follow-up research. As a recent science which is developing rapidly under the growing attention that the field of Environmental Health is attracting, human biomonitoring (HBM) is a case in point. Both its overlap of the fields of medicine and environmental sciences and the difficulty in categorically classifying it as any of the classical epidemiological study designs, further mean a lack of comparable experience and correspondingly, of any existing legal framework.

In this regard, it is the authors' objective to disclose the main ethics issues encountered during human biomonitoring conducted in Portugal and describe how they have been handled and/or solved.

## Methods

The authors' current biomonitoring projects, hereby referred to by their Portuguese acronyms, include ProVEpA, which involves biomonitoring of dioxin-like compounds and heavy metals, in tissues including blood and pubic hair, in the general population and pregnant women; monitoring of lead and mercury in blood and hair in children; and monitoring of dioxin-like compounds in human milk, and having involved approximately 1,700 participants in both Lisbon and Madeira since 1999. Its main aim is the surveillance of the effects of the proximity of municipal solid-waste incinerators on levels of the potentially most harmful substances released during the process of incineration. Temporal trends of these levels are also monitored, as are potential adverse health effects in neighbouring populations.

Recruitment strategies have evolved since the start of the study. Currently, general population adults are recruited among blood donors through collaboration with the national institute for blood collection which allows the authors' team to accompany its mobile units. Pregnant women are recruited at ante-natal appointments in maternities or, when umbilical cord blood and pubic hair samples are required as opposed to venous blood, in pre-labour in obstetric wards. They are often followed through and further donate human milk samples. Recruitment of children aged one to six years is performed in hospitals and primary health-care centres. For blood sampling, targeted children are those scheduled to draw blood for other purposes, whereas for hair sampling a hair-cut is purposely arranged.

In all of these groups recruitment is only conducted among apparently healthy individuals and equal numbers of age and (when relevant) gender-paired residents or workers in the areas of study and control areas are enrolled. For children, age groups are targeted in order that ages 12–24 and 25–36 months are 50% over-represented as compared to older groups.

The project entitled VAEDA involves the monitoring of blood lead and dioxin-like compounds and their potential association with the incidence of miscarriage and aims to enrol 200 women in Lisbon and Madeira. The recruitment strategy of VAEDA involved setting up a toll-free call centre and widely disseminating details of the study including its phone number, in order to reach the specific target population of women trying, or about to try, to become pregnant. Dissemination has comprised TV reports and interviews, articles in newspapers and magazines, raising awareness among primary health care workers and the distribution of flyers and posters in primary health-care centres, maternities, pharmacies, shops and hairdressers.

Following a World Health Organization (WHO) protocol [3], another study monitors twelve persistent organic pollutants in human milk and geographically representative sampling is being achieved through local recruitments by family health doctors in primary health-care centres.

The authors' most recent study is FEXHE-BIO, determining foetal exposure to lead and correlation with environmental indicators including lead in moss and topsoil. Here recruitment of apparently healthy pregnant women resident in Central Portugal for at least 5 years is ensured by a national network of hospitals and maternities.

In all studies participation involves a tissue or fluid collection procedure, accompanied by a questionnaire and a written informed consent form. It is generally a once-off event, with the only exceptions being annual sampling performed for general population adults in Madeira, and daily urine collection during three menstrual cycles for women attempting pregnancy in VAEDA. No medical records are consulted and all requested health information is provided by the participant, of their own free will. Written informed consent is obtained in all studies conducted. It includes a brief explanation of aims, benefits and requirements of participation, an explanation that withdrawal of consent is permitted at any time and an assurance of the confidentiality of data collected.

Results are communicated to participants only when their readings are outside the normal range towards levels considered adverse to health, with the exception of pregnancy monitoring in VAEDA, whose results will be imparted at the end of the study.

Compensation is generally not provided in return for participation. The exceptions are, for participants in the ProVEpA project, the provision of routine blood tests to general population adults and of vouchers for hair-cuts to children, which simultaneously serve the purpose of hair sampling. Participants in VAEDA are offered the opportunity to participate in a number of health awareness actions.

## Results and discussion

### Autonomy

Autonomy is one of the basic ethical principles governing all research involving human subjects. It upholds the subjects' right to be fully informed and free to decide whether they wish to participate [4] and underlies the requirement for situated Informed Consent. However, groups with diminished autonomy, such as children, though impeded from providing consent in these terms, cannot ethically be excluded from research, as often they are among the most vulnerable and at-risk groups and as such are afforded specific protection under national law [5]. The responsibility for the protection of their rights is shifted to their guardians, with the researchers also accountable through their code of ethics.

Increasingly, in research involving children, the assent of the child is required, in addition to the informed consent of one or both parents/guardians. This involves a child's affirmative agreement to participate in the research and not mere failure to object [6]. Although assent of the child is not explicitly defined or required by law, their rights are assured by national law [7,8] and the European Convention on Human Rights and Biomedicine determines, in the context of health interventions, that the opinion of the minor must be taken into consideration in proportion to their age and maturity [9]. Children, during gestation and after birth, form an important component of the authors' research. This results from a decision implicitly prioritizing the protection of a vulnerable group over their right to self-determination.

The afore-mentioned Convention also defines that research may only be carried out on a person who does not have the capacity to consent if "the results of the research have the potential to produce real and direct benefit to his or her health" unless "the research has the aim of contributing, through significant improvement in the scientific understanding of the individual's condition, disease or disorder, to the ultimate attainment of results capable of conferring benefit to the person concerned or to other persons in the same age category or afflicted with the same disease or disorder or having the same condition"[9]. Although the latter validates research activities in biomonitoring, surveillance is not encompassed in the definition of research as the production of new, generalizable knowledge, as distinct from knowledge pertaining only to a particular individual or programme [10]. However this borders on a semantic issue in that surveillance equally aims to contribute to the scientific understanding of individuals' condition, with the ultimate aim of conferring benefit to the person concerned or others in the same condition by providing information which can be linked to health effects. As such, it is the authors' best interpretation that surveillance should be guided by this legislation in addition to pondering input from the relevant health ethics committees and other bodies.

### Beneficence

The principle of doing good [2,4], or maximizing benefits over harms [10] and non-maleficence, the principle of not harming, inherent to the field of health-care, can become a surprisingly contentious issue in public health, in that the latter is principally concerned with community health, whose interests occasionally collide with the interests of health or well-being of individuals. An interesting example is vaccination: in diseases which are nearly eliminated, incidence of adverse effects is in some cases greater than morbidity of the disease itself, yet elimination is obviously in the public's interests.

Biomonitoring is even more debatable in that direct health benefits are only occasionally produced, for example when levels detected are in the range of those known to affect health and remedial action is possible. Where biomonitoring involves research into the health effects of a substance's body burden, health benefits are indirect and not immediate.

Invasive sampling techniques such as blood withdrawal pose a small but not insignificant risk to an individual's health, which in the context of research is generally judged to be outweighed by the greater community good.

In the context of children's participation, requesting altruistic actions from an individual who cannot provide true informed consent is ethically debatable however, notwithstanding the nobility of the principle of protecting the vulnerable.

In legislative terms, the situation is equally contradictory, with the European Convention on Human Rights stating that "the interests and welfare of the human being shall prevail over the sole interest of society or science", yet foreseeing restrictions to this "in the interest of public safety, for the prevention of crime, for the protection of public health or for the protection of the rights and freedoms of others" [9].

The authors' current research includes attempts to correlate hair and blood heavy metal levels in children, to determine if the less invasive technique with hair samples could replace the need for blood sampling. Similarly, pubic hair from pregnant women at the time of birth is collected and analysed for correlation with blood heavy metal levels.

### Other ethics issues related to recruitment and sample collection

The lack of any immediate benefit for the participant, while not in itself a deterrent, does not on the other hand constitute an incentive to voluntary participation. Added to the fact that individuals should not be pressured to participate in a study [10], recruitment can often constitute a limiting factor. Whereas payment for participation is often deemed unethical and may even be illegal [9], "compensation" for time lost or travel expenses is acceptable practice. Our experience of performing and reporting routine blood tests as a bonus has proven successful in the ProVEpA project in recruiting participants from the general population. Refusals to participate are extremely rare in this group, mainly due to the fact that recruitment is currently performed among blood donors and participation implies only a small increase in the volume of blood withdrawn. The strategy of approaching subjects who are drawing blood for other purposes has also proven successful in recruiting pregnant women, in both ProVEpA and FEXHE-BIO, with few refusals reported.

Recruitment of children has been problematic however, and medical appointments have not proven to be successful recruiting-ground, not only because the children are frequently accompanied by an adult other than a parent, who are understandably less willing to provide consent, but also because the parents themselves will often not allow blood withdrawal solely for monitoring purposes if it is not otherwise required for medical reasons. Obtaining hair samples from the children has posed the least problems and the compensation strategy of providing vouchers for corrective hair-cuts has proven popular. An initial attempt at recruiting children from pre-school facilities was abandoned after, in addition to parents' reticence, staff expressed concerns about the children associating the negative experience of blood withdrawal with the location, aggravating the unwillingness many children already displayed in attending. This was upheld as a valid ethical concern.

Consent has also not been the obstacle to obtaining pubic hair from pregnant women as they are recruited in pre-labour in obstetric wards prior to routine pubic shaving. Differences in grooming habits between regions have led to women in Lisbon providing insufficient hair for these analyses to continue while in Madeira this problem has not arisen and sampling is still in place.

Donation of umbilical cord blood for sampling in ProVEpA provides useful estimates of maternal pollutant load and of foetal exposure, but decreases in adhesion rates forced the practice to be dropped in Lisbon, although still ongoing in Madeira. A contributing factor is thought to be the increase in the number of parents cryo-preserving umbilical cord stem cells in private bio-banks for future health-care options for their child, an interesting ethical issue itself [11].

The non-targeted recruitment strategy of VAEDA, where potential participants contact a toll-free call centre of their own initiative, enables records of contacts made and subsequent participation to be kept, with a current recruitment rate of 44%. The main reasons for not participating include non-eligibility and misunderstanding of study aims. The strategy also guarantees a low refusal rate – just 13% of women who contact the call-centre but do not end up participating.

Health-care worker involvement in recruitment has proven one of the most successful strategies, in both VAEDA and FEXHE-BIO, due to their direct contact with the pool of potential participants which means a greater number can be approached and also to the professional relationship of trust, which improves rates of consent.

Media participation in publicizing the projects has been invaluable in studies such as VAEDA which involve recruitment addressed to an extremely specific population sub-group (healthy women attempting to become pregnant). A broader dissemination of the aims and results of HBM needs to be undertaken as a priority, not only as raising public awareness should increase participation rates, but also in the interests of openness and transparency.

### Conflicts of interests

Any agency performing independent intervention evaluation/consultancy is faced with a potential conflict of interest. ProVEpA is conducted as a surveillance study of human exposure to the most critical pollutants of solid-waste incinerators and potentially associated health effects in populations residing in their vicinities, commissioned by waste management companies Valorsul and Valor Ambiente. The study is therefore financed by entities that have obvious vested interests in the outcome of the study. Ethically, transparency is key, requiring full disclosure of the conflict of interest [10] and relying on the scientific objectivity and impartiality of the researchers and their responsibility to publish findings, independent of possible negative reactions from any stakeholders, whether they are public, governmental or industrial [12]. In parallel, a supervisory committee appraises the rigour and independence of the study and of the contactor-consultancy relationship.

The waste management companies are required, as a follow-up to environmental impact reporting conducted as a part of the licensing procedure, to carry out Environmental and Public Health monitoring programs, as risk surveillance and control measures, for the duration of the facility's life span. This practice was compulsorily implemented in the late nineties in response to public and scientific concerns over the widespread use of incineration, in an attempt to mitigate the conflict of interests between the public's right to the safeguarding of their health and to the proof thereof, and the companies' interests. However, as with other forms of public health research, evaluation of results requires careful study of risk-benefit ratios. Waste incineration is conducted in the public's interests and is not a purely profit-making industry. Potential negative impacts of waste incineration on human health must be weighed against the relative risks to both human health and environmental quality of alternative methods of waste disposal and not simply against its absence.

### Information practices

Obtaining informed consent is crucial to safeguard the autonomy of the participants and is obtained in written form in all studies conducted. It also constitutes an information practice since it usually includes a brief explanation of aims, benefits and requirements of participation, an explanation that withdrawal of consent is permitted at any time and an assurance of the confidentiality of data collected. It covers sample collection and handling and storage and processing of personal data. Consequences and risks are not generally specified as they are only relevant in the case of blood sampling, and in this case (with the exception of VAEDA) the participants are drawing blood irrespectively so possible negative consequences are not linked to, and as such do not constitute a deterrent from, participation.

Assent is not obtained from children as a rule due to their age (one to six years) – in keeping with European law which states that the opinion of the minor shall be considered in proportion to their age [9] -, and to their drawing blood for other purposes. Full written informed consent is invariably required from a parent/guardian.

Besides informed consent prior to the study, reporting of results also presents another occasion for information to be made available to participants. The 'right to know' concerning an individual's health information has lately been supplemented by the 'right not to know', of particular interest when the health effects of a substance are not know, or if known, reduction options are not clear. Both these rights are protected by European Convention [9]. Current participants in ProVEpA, FEXHE-BIO and the human milk survey are not asked if they wish to be informed of their results and are only individually informed if their readings are outside the normal range, in which case they are informed through their family doctor, in keeping with the law [13]. VAEDA participants will be fully informed of their results – incidence of miscarriages and blood lead and dioxin load – at the end of the project, and the authors have found this to be the main incentive for participation, but similarly an information opt-out is not explicitly offered.

Communicating or not the dioxin or heavy metal load in human milk (ProVEpA and human milk survey) is a sensitive issue which must be approached particularly carefully. The benefits of breast-feeding are so significant that the WHO has advised that they outweigh any risks resulting from exposure of the nursing child to raised pollutant levels in milk [14]. This must be unambiguously communicated to mothers when informing them of their raised pollutant load so that their concerns for the health of their infant do not lead them to stop breast-feeding. The fact that human milk samples and pubic hair samples are frequently pooled for analysis (due to lack of sufficient quantity for individual analysis) further complicates matters (human milk survey and ProVEpA in Madeira), as here abnormal values must be reported highlighting the degree of uncertainty due to the multiple and unknown contributions of the group elements.

Informed consent forms explicitly state that samples will only be kept until analysis and no use beyond that initially predicted and informed to the donor will be made.

### Data protection

Data protection is another topic where public interests are put above the rights of the individuals, namely the right to privacy [4]. Maintaining data confidentiality is a current public concern raised, possibly unduly, by increased use of computerized databases. This topic has been gaining ever greater attention from researchers and law-makers. In spite of this, there is still a lack of regulation on specific aspects in many countries, including Portugal, as can be seen from the tables on data protection, presented in the document "Ethics and data protection in a number of European countries" [15], compiled within the ESBIO project [16].

In order to be able to communicate individual results, and to guarantee access to participants for follow-up sampling in VAEDA and in the human milk survey, volunteers' identities can not be irreversibly anonymized. However, as all projects store sensitive data on health, participants' names are coded prior to data processing and analysis and database access is protected by user passwords, conforming to legislation on information storage [13]. The security and confidentiality of the data stored is ensured by a number of measures designed to restrict and/or register all forms of access to participants' personal data including physical access to facilities, access to databases and other means of storage and access to means of data insertion and processing [17].

Besides covering all aspects of sample collection, informed consent sought from participants also foresees both the storage and processing of data concerning them. The guarantee of the confidentiality of this data under any circumstances is a fundamental part of initial information practices.

### Ethics committees

Although no official opinions from national ethics bodies have been stated in regard to human biomonitoring [15], all Portuguese public health institutions have an ethics committee to which research and surveillance projects using a HBM approach have to be submitted prior to their commencement, as any project involving data and sample collection. All study protocols mentioned were approved by the University of Lisbon, Faculty of Medicine Ethics Committee. In addition, projects where recruitment involved collaborations with other health institutes were also approved by their respective ethics committees, as was the case of Dr Alfredo da Costa Maternity where pregnant women are recruited in Lisbon and Dona Estefânia Hospital where children were formerly recruited, and all the hospitals and maternities collaborating with FEXHE-BIO's aims of determining foetal exposure to lead. Appraisals from health ethics committees are not legally binding [18] but it is accepted practice to conform to the recommendations made.

### Current legislation and constraints

Apart from legislation covering collection of health information [13], data protection [17], ethics committees [18], the basis of national health policy [5] and individuals' rights [7,8], the only legislation regulating scientific research involving humans not classified as clinical trials is the European Convention on Human Rights and Biomedicine ratified to national law [9]. In addition, no national legislation currently accounts for the surveillance component of biomonitoring as distinct from research, so ethical issues including those presented here are frequently left to the best judgement of the involved professionals or ethics committees.

## Conclusion

The authors are satisfied that the issues of autonomy and beneficence are being handled according to ethical principles, in spite of legislative uncertainty. Current information practices require some alterations, particularly as to the provision of the right to know. Potential misinterpretation of the concept of biobanking in regard to sample storage for the relatively short period between collection and analysis means sample storage practices within the projects underway probably ought to be re-evaluated according to Article 19 of Law no. 12/2005, dated January 26^th ^[13]. As the most conspicuous ethical issue, the presence of conflicts of interest has been successfully handled. Raising the profile of human biomonitoring – which the upcoming European-scale Pilot Project to develop a coherent approach to Human Biomonitoring in Europe, foreseen under the European Commission's Environment and Health Action Plan 2004–2010 [19], will contribute to – could have important consequences in terms of public awareness, contributing to improved recruitment rates, and also in legislative terms, thereby clarifying and validating HBM's standing.

## Competing interests

The authors declare that they have no competing interests.

## Authors' contributions

MFR and JPM designed, organize and/or coordinate all projects referred to. AB and MM recruit participants and perform technical and laboratory work for ProVEpA; SS, HTS and MM for VAEDA; VG for FEXHE-BIO; and AB for the human milk project. MFR and SS are responsible for the manuscript text.
